# Anästhesie bei Aortenklappenstenose: Anästhesiologisches Management von Patienten mit Aortenklappenstenose bei nichtkardiochirurgischen Eingriffen

**DOI:** 10.1007/s00101-024-01380-x

**Published:** 2024-02-09

**Authors:** Sebastian Billig, Marc Hein, Moritz Uhlig, David Schumacher, Marcus Thudium, Mark Coburn, Christina K. Weisheit

**Affiliations:** 1 Klinik für Anästhesiologie, Universitätsklinikum Aachen, Pauwelsstr. 30, 52074 Aachen, Deutschland; 2 Klinik für Anästhesiologie und operative Intensivmedizin, Universitätsklinikum Bonn, Venusberg-Campus 1, 53127 Bonn, Deutschland

**Keywords:** Perioperatives Management, Nichtkardiale Chirurgie, Risikoreduktion, Herzklappenerkrankung, Echokardiografie, Perioperative management, Noncardiac surgery, Risk reduction, Valvular heart disease, Echocardiography

## Abstract

Die Aortenklappenstenose ist eine häufige Erkrankung, die dem behandelnden Anästhesisten profundes Wissen über die Pathophysiologie, Diagnostik und die perioperativen Besonderheiten der Erkrankung abverlangt. Eine neu aufgetretene Aortenklappenstenose wird vielfach erst durch klinische Leitsymptome (Dyspnoe, Synkopen, Angina pectoris) bzw. einen auffälligen Auskultationsbefund im Rahmen der anästhesiologischen Prämedikationsvisite entdeckt und erfordert ein interdisziplinäres Management, um die optimale Behandlung der Patienten im perioperativen Setting zu gewährleisten. Für die individuelle Auswahl des Anästhesieverfahrens ist eine präzise Befunderhebung im Rahmen der Prämedikationsvisite erforderlich, und darüber hinaus eine genaue Kenntnis der hämodynamischen Besonderheiten der Aortenklappenstenose. Der folgende Übersichtsartikel führt nach einer kurzen Rekapitulation der allgemeinen Pathophysiologie der Erkrankung durch die anästhesiologischen Besonderheiten, die Risikofaktoren für Komplikationen und das perioperative Management bei nichtkardiochirurgischen Operationen von Patienten mit Aortenklappenstenose.

## Abstract (en)

Aortic valve stenosis is a common condition that requires an anesthesiologist’s in-depth knowledge of the pathophysiology, diagnostics and perioperative features of the disease. A newly diagnosed aortic valve stenosis is often initially identified from the anamnesis (dyspnea, syncope, angina pectoris) or a suspicious auscultation finding during the anesthesiologist’s preoperative assessment. Interdisciplinary collaboration is essential to ensure the optimal management of these patients in the perioperative setting. An accurate anamnesis and examination during the preoperative assessment are crucial to select the most suitable anesthetic approach. Additionally, a precise understanding of the hemodynamic peculiarities associated with aortic valve stenosis is necessary. After a short summary of the overall pathophysiology of aortic valve stenosis, this review article focuses on the specific anesthetic considerations, risk factors for complications, and the perioperative management for noncardiac surgery in patients with aortic valve stenosis.

## Epidemiologie

Die valvuläre Aortenklappenstenose (AS) zählt zu den häufigsten Herzklappenerkrankungen in den Industrienationen und ist darüber hinaus der häufigste behandlungsbedürftige Herzklappenfehler in Deutschland. Bei den über 75-Jährigen liegt die Prävalenz aller Formen der AS bei ca. 12,5 % und die Prävalenz einer hochgradigen AS bei ca. 3,5 %, von denen über 75 % Symptome zeigen [26]. Bei den über 85-Jährigen steigt die Prävalenz der schweren AS auf mehr als 8 % [18]. Frauen und Männer sind in etwa gleich häufig von einer AS betroffen. Da die Prävalenz der Erkrankung mit zunehmendem Alter steigt, darf davon ausgegangen werden, dass die Fallzahlen in den kommenden Dekaden infolge des demografischen Wandels weiter zunehmen werden [34].

## Formen, Pathophysiologie und Symptome

In westlichen Industrienationen ist die führende Ursache der valvulären AS mit über 80 % der Fälle die degenerativ-kalzifizierende AS [14]. Hier kommt es durch Prozesse, die denen der Atherosklerose ähnlich sind, zunächst zu einer Sklerose in der dünnen Endothelschicht der nativen Aortenklappe. In der Initialphase stehen entzündliche Prozesse und eine Lipidinfiltration der Klappe im Vordergrund, die dann im Verlauf in eine Fibrosierung und Kalzifizierung der Klappe übergehen. Die Risikofaktoren für die Entwicklung einer AS sind Alter, arterielle Hypertension, Niereninsuffizienz, Diabetes mellitus, Dyslipidämie und Rauchen. Somit haben die AS und die Atherosklerose ähnliche Risikofaktoren.

Eine bikuspide AS ist die häufigste Ursache der AS bei den unter 70-Jährigen und der häufigste klinisch relevante kongenitale Herzfehler [21, 34]. Durch die unphysiologischen Strömungsverhältnisse entstehen Scherkräfte, die zu einem erhöhten Verschleiß der Klappe führen; dies entspricht näherungsweise einem akzelerierten Verlauf der degenerativen AS. Daher stellt eine durch eine bikuspide Aortenklappe bedingte AS auch die Indikation für ca. 50 % der herzchirurgischen Aortenklappenersatzoperationen dar.

Durch den weit verbreiteten Einsatz von Antibiotika spielt die postrheumatische AS in Industrieländern heutzutage nur noch eine untergeordnete Rolle. In Entwicklungsländern ist die Inzidenz jedoch weiterhin hoch. Hierbei kommt es durch immunologische Phänomene zu einen Umbau der Herzklappen, typischerweise voranging der Mitralklappe, aber auch der Aortenklappe.

Bei allen Formen der AS führt die eingeschränkte Beweglichkeit der Klappe in einem langsam voranschreitenden Prozess zu einer Obstruktion des linksventrikulären Ausflusstrakts. Zur Kompensation der konsekutiven linksventrikulären Nachlasterhöhung kommt es zu einer konzentrischen Hypertrophie und Fibrose, wodurch die Wandspannung trotz der erhöhten Nachlast konstant bleibt [6]. Diese Umbauprozesse führen zu einer Versteifung und somit zu einer eingeschränkten Compliance des Ventrikels. Des Weiteren führt die Abnahme der relativen Kapillardichte (konstante Zahl der Kapillaren bei Zunahme der Muskelmasse) zu einer eingeschränkten myokardialen Perfusionsreserve [20]. Bereits geringe Steigerungen der kardialen Last können daher bei schon in Ruhe erhöhter Schlagarbeit die Perfusionsreserve ausschöpfen und den bestehenden Sauerstoffmangel des Ventrikels aggravieren [8]. Auch in Abwesenheit einer koronaren Herzkrankheit (KHK) führen diese Mechanismen zu einem erhöhten Risiko für perioperative myokardiale Ischämien. Bei bis zu 70 % der Patienten mit einer AS liegt zusätzlich eine KHK vor, wodurch das Ischämierisiko zusätzlich steigt [16].

Geschlechtsabhängige Unterschiede in der Pathophysiologie der AS sind Gegenstand aktueller Forschung und bisher unzureichend verstanden. Dennoch kann festgehalten werden, dass Frauen zu einer diffusen Fibrose des Herzmuskels mit eingeschränkter Pumpfunktion bei konzentrischer Hypertrophie tendieren, während Männer eher zu einem exzentrisch dilatierten Ventrikel neigen [13].

Die AS ist eine über Jahr(zehnt)e langsam fortschreitende Erkrankung von Herzklappe und Herzmuskel, die lange klinisch inapparent bleiben kann [27]. Als erste Anzeichen können sich unspezifische Symptome wie eine belastungsinduzierte Dyspnoe oder Angina pectoris (AP) zeigen. Typische Symptome wie Synkopen mit ggf. rezidivierenden Stürzen, Schwindel, Fatigue bis hin zu AP in Ruhe zeigen sich erst im Endstadium der Erkrankung im Rahmen einer hochgradigen AS [25]. Insgesamt sind ca. 54–66 % der hochgradigen symptomatischen AS nicht diagnostiziert [35]. Mit Beginn der Symptome steigt die Letalität ohne Operation rapide an und bringt eine Einjahressterblichkeit von ca. 30 % mit sich [31].

## Diagnostik

**Figure 1:**
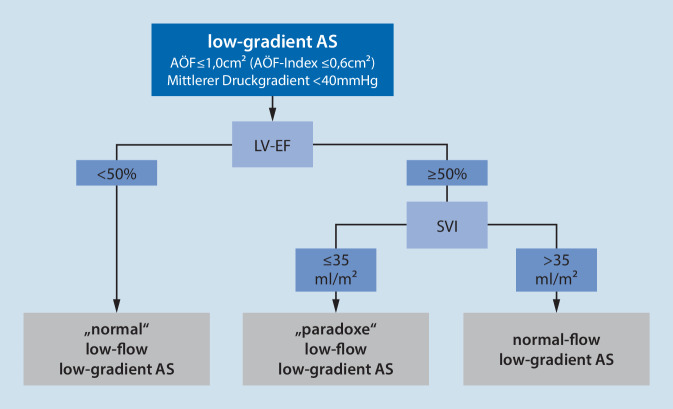

Nachdem sich der Verdacht einer neu aufgetretenen AS aus Anamnese oder körperlicher Untersuchung ergeben hat (Tab. 1), ist das zentrale Instrument für die Diagnostik und Klassifikation der AS die transthorakale Echokardiographie (TTE). Anhand der mittels Kontinuitätsgleichung ermittelten Klappenöffnungsfläche (KÖF), der maximalen systolischen Flussgeschwindigkeit über der Aortenklappe und dem mittleren Druckgradienten kann die AS in eine leichte, mittelgradige und schwere Form eingeteilt werden (Tab. 2). In diesem Zusammenhang sollte dem Anästhesisten auch die „low-flow, low-gradient“ AS geläufig sein: Durch eine begleitend auftretende Herzinsuffizienz bei der AS ist der linke Ventrikel nicht in der Lage, ein adäquat hohes Schlagvolumen auszuwerfen, sodass nur ein geringer mittlerer Druckgradient über der stenosierten Aortenklappe vorliegt ([4, 36]; Abb. 1). Diese Form macht bis zu 35 % der Fälle unter den schweren AS aus [4]. Zu beachten ist auch, dass eine systemische Hypertension den Druckgradienten über der stenosierten Klappe absenken kann. Die Interpretation der TTE-Befunde hat daher immer in Zusammenschau mit den weiteren Befunden stattzufinden. Mithilfe eines Dobutamin-Stress-Echos in niedriger Dosierung kann diese Form „enttarnt“ werden. Da v. a. Frauen zu einer konzentrischen Hypertrophie tendieren, sind sie auch häufiger von der „low-flow, low-gradient“ AS betroffen als Männer [13].

**Table 1:** 

Diagnostikum	Beschreibung
Anamnese	Eingeschränkte Belastbarkeit, Dyspnoe, Angina pectoris, Fatigue, Schwindel, Synkope. Einteilung einer Herzinsuffizienz nach der Klassifikation der New York Heart Association (NYHA). *Cave*: Durch Vermeidungsverhalten berichten Patienten fälschlicherweise oft, keine Belastungssymptomatik zu haben [29]
Körperliche Untersuchung	Auskultation: lautes Systolikum im 2. Interkostalraum rechts parasternal, leiser 2. Herzton. Klinische Zeichen einer Herzinsuffizienz: erhöhter Jugularvenendruck, Pulsus tardus et parvus, Tachykardie, pulmonale Rasselgeräusche, periphere Ödeme
EKG	Positiver Sokolow-Lyon-Index, (überdrehter) Linkstyp, T‑Negativierungen V_4_–V_6_
Echokardiographie	Tab. 2
Kardio-MRT, Herzkatheteruntersuchung, Stressechokardiographie	Nur in Spezialfällen und nach kardiologischer Rücksprache

**Table 2:** 

	Leichte AS	Mittelgradige AS	Schwere AS
*AÖF [cm*^*2*^*]*	> 1,5	1,0–1,5	≤ 1,0
*AÖF-Index [cm*^*2*^*/m*^*2*^*]*	> 0,85	0,6–0,85	≤ 0,6
*Jetgeschwindigkeit [m/s]*	< 2,9	3,0–4,0	≥ 4,0
*Mittlerer Druckgradient [mmHg]*	< 20	20–40	≥ 40

*AÖF* Aortenklappenöffnungsfläche

Eine weitere Form der „Low-flow-low-gradient“-AS ist die „paradoxe Low-flow-low-gradient-AS“ oder „Low-flow-low-gradient-AS mit erhaltener EF.“ Ein kleines Cavum (mit dann normaler EF) kann kein adäquates Schlagvolumen generieren, sodass der resultierende Druckgradient entsprechend geringer ausfällt [36]. Eine Mitralklappeninsuffizienz kann ebenfalls zu dieser Form der AS führen. Die dritte Form der Low-gradient-AS ist die „Normal-flow-low-gradient-AS“, bei der der reduzierte Gradient über der AS durch eine Bradykardie bzw. Hypertonie erklärt werden kann.

**Figure 2:**
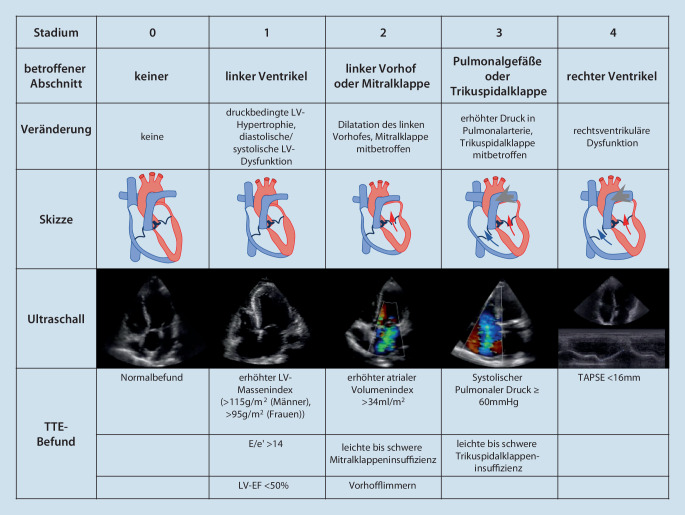

Die AS kann darüber hinaus, basierend auf der hämodynamischen Erkrankungsschwere und dem Einfluss der AS, bezüglich des sekundären myokardialen Schadens klassifiziert werden ([9]; Abb. 2). Durch die AS können zunächst eine Linksherzinsuffizienz sowie eine Mitralklappeninsuffizienz entstehen. Durch eine sekundäre pulmonale Hypertonie kann es im finalen Stadium zu einer Affektion des rechten Ventrikels kommen.

Kardiale Biomarker wie B‑type Natriuretic Peptide (BNP) oder Troponin haben in den letzten Jahren zunehmend Aufmerksamkeit in der Diagnostik, Prognoseabschätzung und Therapiesteuerung der AS erhalten und werden mittlerweile auch für die präoperative anästhesiologische Risikostratifizierung empfohlen [10, 28]. Das BNP bzw. sein Prohormon NTproBNP werden in Vorhof und Ventrikel auf einen Dehnungsreiz hin freigesetzt und zeigen eine erhöhte Wandspannung an, die auf eine hämodynamische Relevanz der AS hinweisen kann. Troponinerhöhungen hingegen weisen auf einen Zelluntergang im Rahmen ungünstiger Umbauprozesses des Ventrikels hin. Das BNP und Troponin können ebenfalls zur Prognoseabschätzung im Vorfeld einer Transkatheter-Aortenklappenimplantation (TAVI) genutzt werden [30].

## Therapie

Für die AS gibt es bisher keine medikamentösen Therapieoptionen, die den Progress der Erkrankung verlangsamen oder sogar umkehren könnten. Daher beschränkt sich die Behandlung auf die Symptomkontrolle bzw. die Behandlung evtl. begleitend auftretender Erkrankungen wie z. B. einer arteriellen Hypertonie. Bei Patienten mit schwerer AS ist die Therapieempfehlung abhängig vom Vorhandensein von Symptomen und dem periinterventionellen Risiko. Besteht eine Indikation zum Klappenersatz, sollte ein interdisziplinäres Herz-Team involviert werden. Die definitive Versorgung kann interventionell mittels TAVI oder eines offen kardiochirurgischen Klappenersatzes erfolgen [36]. Ohne Symptome sind weitere Parameter, wie der Aktivitätsstatus der Patienten und die linksventrikuläre Pumpfunktion, in die Entscheidung zur Therapieempfehlung für den Patienten miteinzubeziehen [36]. Die Ballonvalvuloplastie ist aufgrund der hohen Restenose- und Komplikationsrate keine definitive Therapieoption und sollte nur in Einzelfällen als überbrückende Intervention bis zur definitiven Therapie erwogen werden [10].

## Präoperative Evaluation

Ziel der präoperativen anästhesiologischen Beurteilung ist es, die relevanten Vorerkrankungen der Patienten zu erfassen und zu beurteilen sowie Patienten mit einem mittleren und hohen perioperativen Risiko zu identifizieren. So kann eine adäquate Risikostratifizierung vorgenommen werden und, sofern möglich, eine zielgerichtete präoperative Optimierung des Patientenzustandes stattfinden.

Folgende Schritte sollten bei der präoperativen Evaluation bei Patienten mit bekannter oder vermuteter AS durchgeführt werden:

- Anamnese und körperliche Untersuchung mit besonderem Fokus auf die Zeichen einer AS bzw. Herzinsuffizienz (Tab. 1),
- Diagnosesicherung mittels TTE bei vermuteter AS bzw. Beurteilung des Schweregrads unter besonderem Augenmerk auf den mittleren Gradienten sowie die links- und rechtsventrikuläre Funktion bei bekannter AS,
- EKG-Erfassung und -Beurteilung,
- Bestimmung kardialer Biomarker (Troponin und BNP),
- Screening auf eine konkomitante KHK und andere kardiovaskuläre Risikofaktoren.

Bei bekannter AS ist der natürliche Progress der Stenose zu beachten: Es kann davon ausgegangen werden, dass der mittlere Gradient über der Klappe um ca. 5–15 mmHg/Jahr zunimmt und die KÖF um ca. 0,1 cm^2^/Jahr abnimmt [24]. Die letzte echokardiographische Untersuchung vor der Operation sollte nicht länger als 12 Monate zurückliegen [10].

Das perioperative Risiko von Patienten mit AS ist abhängig von der Symptomatik, dem Schweregrad sowie begleitenden kardiovaskulären Erkrankungen (Tab. 3). Patienten mit einer symptomatischen AS haben ein erhöhtes Risiko für ein unerwünschtes perioperatives kardiovaskuläres Ereignis („major adverse cardiac event“, MACE) bis zu 19 % [33] und eine erhöhte perioperative Letalität. Außerdem wird das Risiko von der Art und Dauer des Eingriffs bestimmt (Tab. 4). Kleinere Eingriffe in Lokalanästhesie bergen ein geringeres Risiko. Ein erhöhtes perioperative Risiko sollte im Anästhesieaufklärungsgespräch mit dem Patienten besprochen werden, entsprechend dokumentiert und in weiterer Folge auch mit der chirurgischen Fachdisziplin kommuniziert werden („shared-decision process“).

**Table 3:** 

Erhöhtes perioperatives Risiko bei Aortenstenose
Schwere AS
Symptomatische AS
Eingeschränkte LV-Pumpfunktion
Mitralklappeninsuffizienz
KHK
Notfalleingriffe

**Table 4:** 

Niedriges Operationsrisiko: < 1 %	Mittleres Operationsrisiko: 1–5 %	Hohes Operationsrisiko: > 5 %
Mammaoperation Zahnbehandlung Augenoperation Schilddrüsenoperation Kleinere urologische/gynäkologische/orthopädische Operationen Kleinere videoassistierte Lungenresektion (VATS)	Karotisoperation, endovaskulärer Aortenaneurysma-Repair HNO-Chirurgie Viszeralchirurgie: Splenektomie, Hiatushernien-Repair, Cholezystektomie Perkutane Angioplastie Nierentransplantation Neurochirurgische Operationen Größere urologische/gynäkologische/orthopädische Operationen	Nebennierenresektion Größere Gefäßchirurgie, Revaskularisierung bei Gefäßverschlüssen untere Extremität, Karotisoperation bei Symptomen Leber- und Pankreaschirurgie, Ösophagektomie Lungen- und Lebertransplantationen Pneumektomie Radikale Zystektomie

**Figure 3:**
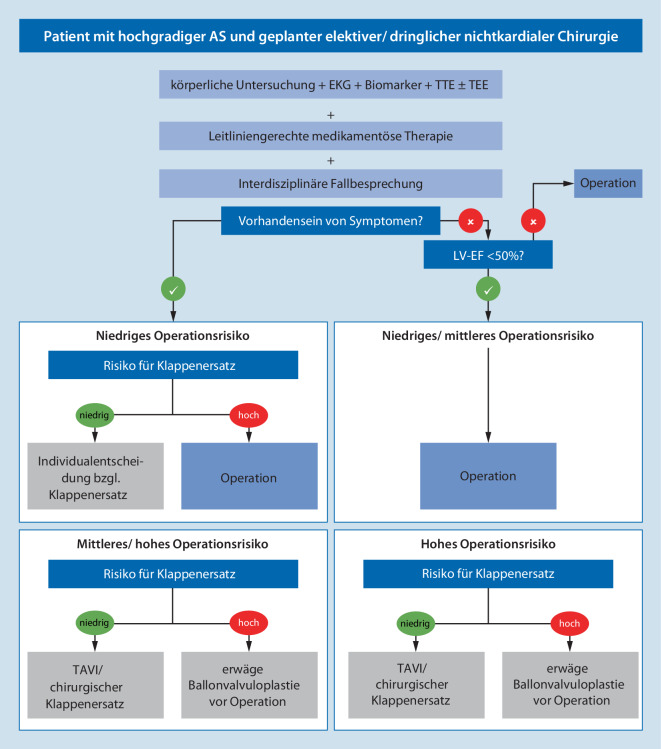

Das anästhesiologische Management sollte sich daher an der Dringlichkeit der geplanten Operation, dem Symptomstatus der Patienten sowie dem eingriffsspezifischen Risiko orientieren [10]. Bei elektiver bzw. nichtdringlicher nichtkardialer Chirurgie ist folgendes Vorgehen empfohlen:

- Bei asymptomatischen Patienten mit schwerer AS und ohne linksventrikuläre Dysfunktion können elektive nichtkardiale Operationen nach einer entsprechenden Vorbereitung durchgeführt werden (Abb. 3).
- Bei symptomatischen Patienten bzw. asymptomatischen Patienten mit eingeschränkter LV-Funktion kann die Indikation für einen vorangehenden Ersatz der Aortenklappe bestehen (Abb. 3; [10]). Diese Patienten können von einem Aortenklappenersatz vor der elektiven Operation profitieren, da dies mit einer geringeren Inzidenz für die Entwicklung einer Herzinsuffizienz, eines Myokardinfarkts, einer ventrikulären Arrhythmie oder eines Schlaganfalls sowie mit einem verbesserten Gesamtüberleben verbunden ist [12]. Aufgrund einer mangelnden Evidenz ist die präoperative Ballonvalvuloplastie stets eine interdisziplinäre Einzelfallentscheidung.

In jedem Fall ist vor der Operation sicherzustellen, dass eine ausreichende Diagnostik erfolgt ist und eine leitliniengerechte medikamentöse Therapie der Begleiterkrankungen durchgeführt wird. Hierzu zählt u. a. die leitliniengerechte Behandlung eines evtl. vorhandenen Vorhofflimmerns, eines arteriellen Hypertonus oder einer Herzinsuffizienz. Bei Patienten mit der Erstdiagnose einer AS im Rahmen der präoperativen Evaluation sollte eine kardiologische Mitbetreuung erfolgen. Zur individuellen Therapieentscheidung bezüglich eines evtl. Klappenersatzes sollte eine interdisziplinäre Fallbesprechung (sofern vorhanden im sog. Heart-Team) erfolgen.

Die zeitliche Dringlichkeit bei Notfalleingriffen wird in den wenigsten Fällen eine vollständige präoperative Evaluation erlauben. In der Literatur gibt es jedoch zahlreiche Hinweise, dass eine fokussierte Echokardiographie durch einen entsprechend qualifizierten Anästhesisten in diesen Fällen die Patientensicherheit erhöhen und das Outcome verbessern kann [2, 3, 11]. Hier sei beispielshaft auf das Curriculum der DGAI zur Ausbildung in der perioperativen fokussierten Echokardiographie verwiesen.

### Begleitendende hämatologische Veränderungen

Patienten mit schwerer AS sind besonders häufig von Anämien betroffen, da sie vielfach eine Behandlung mit Thrombozytenaggregationshemmern und/oder Antikoagulanzien erhalten und zudem an einer erworbenen Koagulopathie leiden können (Von-Willebrand-Syndrom Typ 2A), was zu einem erhöhten Blutungsrisiko führen kann [37]. Die Versorgung des Gewebes mit Sauerstoff kann bereits aufgrund einer verminderten Herzleistung eingeschränkt sein und wird durch eine Anämie weiter eingeschränkt. Für Anämien bei schwerer AS ist bekannt, dass sie den Krankheitsverlauf einer AS negativ beeinflussen können [23]. Aktuell fehlen weiterführenden Studien, die untersuchen, ob ein frühzeitiges Anämie-Management das Outcome verbessert.

## Intraoperatives Management

Die Wahl der Anästhesietechnik basiert auf den Anforderungen des chirurgischen Eingriffs, den patientenindividuellen Komorbiditäten sowie auf den hämodynamischen Spezifika der AS. Grundsätzlich muss bei allen Patienten, bei denen ein anästhesiologisches Verfahren angewendet wird, das Basis-Monitoring aus EKG, Blutdruckmessung und Pulsoxymetrie angewendet werden. Um Myokardischämien zu detektieren, sollte ein 5‑Kanal-EKG mit ST-Strecken-Analyse zur kontinuierlichen Überwachung der Ableitungungen II bzw. einer präkordialen Ableitung (V_4_ oder V_5_) bereits vor der Narkoseeinleitung angebracht werden. Zur kontinuierlichen Blutdruckmessung sollte ein arterieller Katheter vor der Narkoseinduktion in Lokalanästhesie platziert werden. Ein zentraler Venenkatheter kann in vielen Fällen ebenfalls indiziert sein, abhängig von der Erkrankungsschwere der Patienten und dem Risiko der Operation. Auch wenn der zentralvenöse Druck (ZVD) die Flüssigkeitsreaktivität schlecht abbildet, so zeigt er doch eine individuelle Korrelation mit dem Schlagvolumen und kann damit v. a. als Verlaufsparameter hilfreich sein [17]. Der ZVD kann auch genutzt werden, um ein beginnendes Rückwärtsversagens des Herzens (Kongestion) zu identifizieren.

Bei Operationen, die die Zugänglichkeit zum Thorax des Patienten intraoperativ einschränken, kann es sinnvoll sein, Defibrillationselektroden vor der Narkoseeinleitung zu platzieren, um im Bedarfsfall sofort defibrillieren bzw. kardiovertieren zu können. Bei diesen Eingriffen kann auch die transösophageale Echokardiographie von besonderem Nutzen sein, um weitere Informationen über die Pumpfunktion und den Volumenstatus zu erhalten.

Das anästhesiologische Vorgehen bei Patienten mit AS ist in Tab. 5 dargestellt. Diese Maßnahmen sollen sowohl eine globale Minderperfusion als auch eine konsekutive Myokardischämie vermeiden, da dies zu einer weiteren Reduktion der Auswurfleistung und schweren Herzrhythmus-Störungen führen kann.

**Table 5:** 

Parameter	(Patho)physiologie	Anästhesiologisches Vorgehen
*Herzrhythmus und -frequenz*	Der atrialen Ventrikelfüllung kommt bei diastolischer Dysfunktion ein Anteil bis zu 40 % zu, der bei unkoordinierter Aktion von Vorhof und Kammer verloren geht. Tachykardien können durch die Verkürzung der Diastole zu einer eingeschränkten Myokardperfusion führen	Erhalt eines niedrig-normalfrequenten Sinusrhythmus
*Blutdruck*	Eine Hypotension führt zu einer eingeschränkten Myokardperfusion. Eine Hypertension führt zu einem erhöhtem Sauerstoffbedarf des Myokards. Beide Zustände können Herzrhythmusstörungen oder Ischämien bedingen	Erhalt des präoperativen Blutdrucks (Abweichung bis maximal −20 % tolerieren), Narkoseeinleitung ggf. nach Anlage einer kontinuierlichen Blutdruckmessung sowie unter laufendem Norepinephrinperfusor
*Inotropie*	Ein Verlust der Kontraktilität kann den Auswurf des Ventrikels beeinträchtigen und zu einer Dekompensation führen. Eine Steigerung der Inotropie steigert den Sauerstoffverbrauch und kann bei septaler Hypertrophie ggf. eine subvalvuläre Stenose triggern	*Cave*: Bei der Anwendung von stark negativ-inotrop wirkenden Medikamenten (z. B. β‑Blockern). Vorsichtig titrierter Einsatz bei positiv-inotropen Medikamenten (z. B. Adrenalin), ggf. Echokardiographie
*Volumenstatus*	Bei diastolischer Dysfunktion ist eine adäquate ventrikuläre Vorlast zur ausreichenden Füllung der Kammer erforderlich	Einsatz von Echokardiographie zu Evaluation und Optimierung des Volumenstatus. Vermeidung unnötig langer Nüchternzeiten

*Ebenfalls *entscheidend ist das Aufrechterhalten einer ausreichenden zerebralen Perfusion. Als einfach anwendbares kontinuierliches Monitoring hat sich in letzter Zeit die Nah-Infrarot-Spektroskopie (NIRS) etabliert. Auch wenn bisher keine abschließende Evidenz für das Verfahren vorliegt, kann eine optionale Empfehlung für die Anwendung von NIRS abgeleitet werden. Dies ist erst recht der Fall, wenn Komorbiditäten wie Stenosen der A. carotis oder vorangegangene Schlaganfälle vorliegen. Desaturationen > 20 % vom Ausgangswert oder Absolutwerte unter 50 % werden allgemein als kritisch betrachtet und sollten eine Optimierung der hämodynamischen Situation nach sich ziehen [22]. Das prozessierte EEG (pEEG), das hauptsächlich zur Narkosetiefemessung verwendet wird, kann bei konstanter Narkose ebenfalls als Ischämiekriterium mit geringer Sensitivität, aber hoher Spezifität dienen. Da eine Narkosetiefemessung v. a. bei älteren multimorbiden Patienten empfohlen wird, kann das pEEG als ergänzendes Verfahren zu NIRS verwendet werden [5].

### Allgemeinanästhesie

Einleitungsnarkotika und -dosen sollten so gewählt werden, dass die Wahrscheinlichkeit einer Hypotonie minimal ist und gleichzeitig eine ausreichende Anästhesietiefe erreicht wird, um eine sympathische Stimulation und Tachykardie während der Laryngoskopie und endotrachealen Intubation zu vermeiden. Für die nichtkardiale Chirurgie ist sowohl der Einsatz von i.v.- wie auch inhalativen Hypnotika sicher, ohne dass die Wahl des Hypnotikums einen Einfluss auf das postoperativen Outcome des Patienten hat [19]. Aufgrund der bei Ketamin typischerweise einsetzenden Tachykardie sollte von der Narkoseeinleitung mit diesem Hypnotikum eher Abstand genommen werden. Synthetische Opioide wie Sufentanil sorgen für hämodynamische Stabilität während der Induktion. Bei länger dauernden Operationen ist die Gabe einer höheren Opioiddosis während der Einleitung eine Option (z. B. Sufentanil 0,3–1 µg/kgKG). Bei kurzen Eingriffen besteht eine Option in der Gabe des ultrakurz wirkenden Opioids Remifentanil als Ergänzung während und nach der Einleitung in einer Dosis von 1–2 µg/kgKG (oder ≤ 1 µg/kgKG bei älteren Patienten). Remifentanil verringert das Risiko einer Tachykardie, ohne dass das Risiko einer anhaltenden Atemdepression besteht. Eine unmittelbare Verfügbarkeit von Vasopressoren ist sicherzustellen, und zudem sollte das Anästhesieteam über eine ausreichende Erfahrung im Umgang mit kardialen Risikopatienten verfügen. Sofern eine Narkoseeinleitung als Rapid Sequence Induction (RSI) erforderlich ist, kann eine fiberoptische Wachintubation unter entsprechender Abschirmung erwogen werden, um die für die RSI erforderlichen hohen Hypnotikadosierungen zu umgehen.

Zur Aufrechterhaltung der Anästhesie eignet sich sowohl eine totale intravenöse Anästhesie wie auch eine balancierte Anästhesie. Hohe Dosen intravenöser wie auch volatiler Anästhetika sollten wegen der Möglichkeit einer Gefäßerweiterung, Myokarddepression und Hypotonie vermieden werden.

### Regionalanästhesie/neuroaxiale Anästhesie

Sofern möglich sollte bei entsprechend geeigneten Eingriffen stets ein regionalanästhesiologisches Verfahren zur Anwendung kommen, um die Nachteile einer Allgemeinanästhesie oder eines neuroaxialen Verfahrens zu vermeiden.

Bei Patienten mit schwerer AS wird üblicherweise auf eine neuroaxiale Technik verzichtet. Der klassischen Lehrbuchmeinung entsprechend führen das schnelle Einsetzen einer Sympathikolyse und die daraus resultierende Hypotonie dazu, dass diese Patienten den Abfall des koronaren Perfusionsdrucks nicht tolerieren. In der Literatur finden sich jedoch zahlreiche Berichte über erfolgreich durchgeführte Spinal- und Epiduralanästhesien bei Patienten mit schwerer AS ohne Symptome. Insbesondere muss festgehalten werden, dass keine Evidenz für die Unterlegenheit von neuroaxialen Verfahren gegenüber eine Allgemeinanästhesie bei Patienten mit schwerer AS ohne Symptome besteht [15, 32]. Vorteile kann auch die Kombination aus Epidural- und Allgemeinanästhesie bieten. Durch neuroaxiale Verfahren können der Schmerzreiz und somit der kardiale Stress suffizient reduziert werden, und postoperative kardiale wie auch pulmonale Komplikationen können wahrscheinlich reduziert werden [15]. Insofern kann nach entsprechender Risikoabwägung und Aufklärung des Patienten durchaus ein neuroaxiales Verfahren verwendet werden. Wenn eine neuroaxiale Technik gewählt wird, sollten spezifische Vorsichtsmaßnahmen ergriffen werden:

- Beachten einer evtl. bestehenden Antikoagulation bei KHK oder Vorhofflimmern,
- Etablierung einer invasiven Blutdruckmessung vor der Platzierung einer neuroaxialen Nadel oder eines neuroaxialen Katheters,
- langsame Titration (z. B. 3–5 ml alle 5 min) des für die epidurale Verabreichung ausgewählten Lokalanästhetikums,
- Volumengabe zur Aufrechterhaltung eines optimalen intravaskulären Volumenstatus. Die Flüssigkeitszufuhr erfolgt schrittweise (z. B. in Boli von 100–250 ml), um eine Volumenüberlastung bei Patienten mit kongestiver Herzinsuffizienz zu vermeiden,
- umgehende Behandlung einer Hypotonie mit Noradrenalin und ggf. i.v.-Flüssigkeitszufuhr.

## Postoperatives Management

Patienten mit hochgradiger AS haben in der postoperativen Phase ein erhöhtes Risiko, kardiale Komplikationen zu entwickeln, sodass eine intensive postoperative Überwachung indiziert sein kann. Die postoperative Überwachungsstrategie soll sich am Schweregrad der AS und dem eingriffsspezifischen Risiko orientieren [25]. Bei asymptomatischen Patienten mit gering- bis mittelgradiger AS sowie einem maximal mittelgradigen operationsbedingten Risiko kann eine standardmäßige Überwachung im Aufwachraum durchgeführt werden. Bei einer hochgradigen symptomatischen AS oder einem hohen eingriffsassoziierten Risiko empfehlen wir eine Überwachung auf einer Intensiv- oder Zwischenintensivstation. In die Wahl der Überwachungsstrategie sollte auch die Dringlichkeit des Eingriffs einbezogen werden, da Notfalleingriffe mit einer erhöhten Letalität assoziiert sind [33]. Bei Patienten mit schwerer AS, die sich einem nichtkardialen Notfalleingriff unterziehen müssen, ist eine 48- bis 72-stündige intensivmedizinische Überwachung empfohlen [25]. In jedem Falle sollte bei der postoperativen Behandlung darauf geachtet werden, dass eine übermäßige Sympathikusaktivierung durch eine adäquate Analgesie vermieden wird.

## Fazit für die Praxis

- Die valvuläre Aortenklappenstenose hat eine hohe Inzidenz, die mit steigendem Lebensalter zunimmt.
- Auch Patienten mit schwerer Aortenklappenstenose können sich lange asymptomatisch präsentieren, sodass einer genauen Anamnese und Untersuchung in der anästhesiologischen Prämedikationsvisite eine große Bedeutung zukommt.
- Das Vorliegen der folgenden Risikofaktoren erhöhte das perioperative Risiko bei nichtkardiochirurgischen Eingriffen bei Patienten mit Aortenstenose: hochgradige Aortenstenose, symptomatische Aortenstenose, eingetretener myokardialer Schaden (erhöhte Biomarker, reduzierte LVEF, Mitralklappeninsuffizienz).
- Perioperativ sind eine gesteigerte Vigilanz, ein risikoadaptiertes Monitoring, eine Aufrechterhaltung der hämodynamischen Homöostase sowie eine adäquate Überwachung von entscheidender Bedeutung.
- Bei herausfordernden Fällen sollten interdisziplinäre Konferenzen mit Beteiligung der operativen Partner, Kardiologie und Anästhesiologie durchgeführt werden.
